# The impact of fitness social media use on exercise behavior: the chained mediating role of intrinsic motivation and exercise intention

**DOI:** 10.3389/fpsyg.2025.1635912

**Published:** 2025-07-29

**Authors:** Xi Xiao, Dalin Huang, Guanchong Li

**Affiliations:** ^1^School of Sports and Music, Central South University of Forestry and Technology, Changsha, China; ^2^College of General Education, Chongqing Vocational and Technical University of Mechatronics, Chongqing, China; ^3^Institute of Sport Science, College of Physical Education, Southwest University, Chongqing, China

**Keywords:** fitness social media use, exercise behavior, intrinsic motivation, exercise intention, chained mediation

## Abstract

**Objective:**

This study aimed to investigate the psychological mechanisms by which fitness social media use influences individuals’ exercise behavior, focusing on the chained mediating roles of intrinsic motivation and exercise intention.

**Methods:**

A cross-sectional online survey was conducted in April 2025 using snowball sampling, targeting social media users who regularly engage in physical activity. A total of 425 valid responses were analyzed. Constructs including fitness social media use, intrinsic motivation, exercise intention, and exercise behavior were assessed using validated Likert-scale instruments. Structural equation modeling and bootstrap analysis (5,000 resamples) were applied to test the hypothesized chained mediation model.

**Results:**

Fitness social media use was found to significantly predict intrinsic motivation (*β* = 0.396, *p* < 0.001), exercise intention (*β* = 0.254, *p* < 0.001), and exercise behavior (*β* = 0.295, *p* < 0.001). Both intrinsic motivation and exercise intention significantly mediated the relationship between social media use and exercise behavior. The chained mediation pathway was also supported, with indirect effects accounting for 33.55% of the total effect. Specifically, the indirect paths through intrinsic motivation (18.57%), exercise intention (10.75%), and the combined sequence (4.25%) were all statistically significant.

**Conclusion:**

These findings demonstrate that fitness social media use not only exerts a direct effect on exercise behavior but also influences it indirectly through a sequential psychological process involving emotional activation and cognitive planning. The study provides novel empirical evidence supporting the integration of Self-Determination Theory and the Theory of Planned Behavior, thereby offering a deeper understanding of how health behaviors form within digital contexts. In practical terms, this research highlights the significant role of social media–based digital platforms in enhancing intrinsic motivation and exercise intention. Future health-promotion interventions should therefore focus more explicitly on leveraging fitness social media to strengthen individuals’ intrinsic motivation and foster clear behavioral intentions, ultimately facilitating sustained engagement in physical activity and elevating overall population levels of physical activity.

## 1 Introduction

In contemporary society, social media has become deeply embedded in daily life, with its significance and irreplaceable role in information dissemination, interpersonal communication, and cultural construction increasingly recognized (Han and Xu, 2020; Licoppe and Smoreda, 2005; Ohiagu and Okorie, 2014; Sandel and Ju, 2019; Vanden Abeele et al., 2018; Walther, 2017). As of February 2025, 5.56 billion individuals worldwide were internet users, which amounted to 67.9 percent of the global population. Of this total, 5.24 billion, or 63.9 percent of the world’s population, were social media users (Petrosyan, 2025). As public interest in healthy lifestyles continues to grow, social media content centered on fitness, body aesthetics, and nutritious diets has experienced rapid expansion, giving rise to emerging online cultural trends such as “Fitspiration” (Kwon, 2020; Merino et al., 2024; Norton, 2017). An increasing number of users are turning to social media platforms to access exercise-related information, watch fitness tutorials, participate in challenge-based activities, or document their personal workout progress (Bojd et al., 2022; Luhanga et al., 2018; Wang et al., 2024). These practices subtly shape their attitudes and dispositions toward physical exercise (Godin and Shephard, 1990). Among younger populations in particular, social media has become a primary channel for obtaining fitness-related information (Carrotte et al., 2015; Jalali et al., 2020). Research suggests that fitness influencers, internet celebrities, and check-in-style workout content not only effectively stimulate users’ interest in exercise but may also gradually shape their perceptions of a healthy self-image (Durau et al., 2022; Popek, 2024; Wang et al., 2024). Compared with traditional health promotion approaches, the community-oriented and interactive fitness environment fostered by social media is more likely to evoke emotional resonance among users, thereby encouraging behavioral imitation (Rutledge et al., 2024). Therefore, understanding how individuals utilize social media to access fitness information and examining the behavioral consequences of such engagement has become a critical issue within the domains of health psychology and environmental behavior research.

Existing empirical evidence suggests a positive association between fitness-related content on social media and individuals’ engagement in physical exercise (Carrotte et al., 2015; Kim, 2024). The abundance of fitness imagery, training tutorials, and online challenge activities can effectively enhance individuals’ perceived accessibility to exercise-related information, thereby increasing their interest and motivation to engage in physical activity (Prichard et al., 2020). Meanwhile, the unique social interaction features of social media, such as role modeling, self-presentation, and social comparison, may indirectly promote the initiation and maintenance of exercise behaviors by reinforcing individuals’ sense of social identity and behavioral motivation (Tiggemann and Zaccardo, 2015). Some users, through observing and imitating fitness influencers’ workout routines, not only enhance their perceived exercise competence but also gain a sense of group belonging through interactions with community members, which further strengthens their willingness and commitment to sustained physical activity (Durau et al., 2022; Sokolova and Perez, 2021).

In exploring the psychological mechanisms underlying the relationship between social media use and exercise behavior, previous studies have introduced various mediating variables, such as self-efficacy, body image satisfaction, and extrinsic motivation (Ouyang et al., 2021; Ouyang et al., 2020; Tao et al., 2024). However, little is known about the sequential psychological processes through which social media influences exercise behavior via both emotional and cognitive pathways. Most existing research tends to emphasize either broad associations or isolated mediators, offering limited insight into the step-by-step mechanisms that translate online exposure into real-world behavioral engagement. Some studies focus only on the macro-level association between social media use and physical activity (Morningstar et al., 2023), without offering an in-depth analysis of the multilayered psychological processes individuals undergo from information exposure to behavioral execution. This gap in the literature restricts our theoretical understanding of how motivational and intentional factors may operate in tandem within digital health contexts. Moreover, current research has predominantly emphasized external social pressure mechanisms, such as impression management and social comparison (Astleitner and Schlick, 2025; Hauff and Greenleaf, 2022), while giving insufficient attention to internal motivational factors, including personal interest, enjoyment, and autonomous choice. This imbalance has resulted in an underdeveloped account of the intrinsic psychological logic that drives exercise behavior (Brand and Cheval, 2019). Although behavioral intention is widely recognized as a crucial intermediary linking motivation to action (Chu, 2013; Ooi et al., 2011), its specific role within the context of social media use and exercise behavior has yet to be thoroughly modeled and empirically validated. Consequently, the theoretical understanding of individual decision-making processes in this domain remains somewhat ambiguous.

To gain a deeper understanding of the mechanisms through which fitness social media use influences exercise behavior, the present study draws upon Self-Determination Theory (SDT) (Deci et al., 2017; Teixeira et al., 2012) and the Theory of Planned Behavior (TPB) (Conner, 2020) as its theoretical foundation. According to SDT, individuals’ motivation for engaging in specific behaviors can be broadly categorized into intrinsic and extrinsic motivation (Ryan and Deci, 2020). Intrinsic motivation refers to the psychological drive to participate in an activity for reasons such as interest, enjoyment, personal challenge, and internal satisfaction (Deci and Ryan, 2013; Deci and Ryan, 1985). Compared to external incentives, motivation driven by interest and enjoyment has been found to more effectively predict sustained engagement and positive affect toward physical activity (Rodrigues et al., 2020a; Teixeira et al., 2012). Within the context of social media, individuals often experience self-motivating thoughts, such as “I want to try this,” “I enjoy this form of exercise,” or “I want to become stronger,” after viewing training videos posted by fitness influencers, participating in online challenge trends, or encountering other fitness-related content (Dejnaka, 2015; Menhennet, 2025; O'Connor, 2024). These experiences can foster and strengthen intrinsic motivation, thereby promoting a stable and positive inclination toward regular exercise (Gardner and Lally, 2013). While SDT provides a foundation for understanding the emotional and autonomous aspects of exercise behavior, TPB offers a complementary framework that emphasizes the role of cognitive regulation and planned action. The Theory of Planned Behavior further highlights the pivotal role of behavioral intention in bridging the gap between motivation and actual behavior (Godin et al., 2005). According to TPB, the most immediate predictor of behavior is behavioral intention, which is in turn influenced by motivation-related factors, including attitudes toward the behavior, subjective norms, and perceived behavioral control (Barkoukis et al., 2013). In other words, even if an individual possesses sufficient motivation, this motivation must be channeled into clear behavioral intentions, concrete plans, and actionable strategies in order to translate into consistent exercise behavior (Schwarzer, 2008). Previous research has extensively validated the mediating role of behavioral intention in domains such as physical activity (Hagger et al., 2002). In the context of social media, users not only strengthen their intrinsic motivation through content exposure, but also enhance the clarity and enactment of their exercise intentions by documenting their workouts, sharing fitness progress, and engaging in interactive discussions. By integrating SDT and TPB, this study conceptualizes a sequential pathway in which intrinsic motivation, activated by self-directed engagement with fitness content, gives rise to well-formed behavioral intentions that ultimately translate into exercise behavior. This integrative framework allows for a more comprehensive understanding of how emotional drive and intentional planning jointly mediate the influence of social media on physical activity.

Therefore, this study aims to investigate how fitness social media use influences individuals’ exercise behavior through its effects on intrinsic motivation and exercise intention. Based on the above theoretical foundations and research objectives, this study integrates Self-Determination Theory (SDT) and the Theory of Planned Behavior (TPB) to construct a chained mediation model. This model offers a comprehensive perspective on how emotional and cognitive processes jointly mediate the relationship between social media engagement and physical activity.

**Figure 1:**
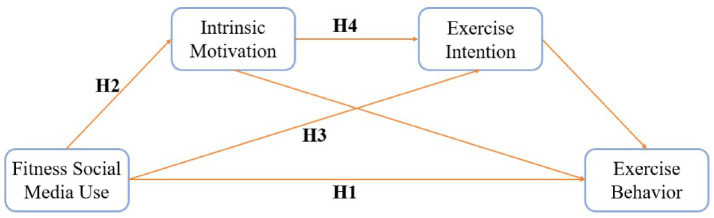
Chained mediation model of the effect of fitness social media use on exercise behavior. Fitness Social Media Use → Exercise Behavior (H1), Fitness Social Media Use → Intrinsic Motivation → Exercise Behavior (H2), Fitness Social Media Use → Exercise Intention → Exercise Behavior (H3), Fitness Social Media Use → Intrinsic Motivation → Exercise Intention → Exercise Behavior (H4).

Accordingly, the following research hypotheses are proposed (Figure 1):

*H1:* Fitness social media use positively predicts individuals’ exercise behavior.

*H2:* Intrinsic motivation mediates the relationship between fitness social media use and exercise behavior.

*H3:* Exercise intention mediates the relationship between fitness social media use and exercise behavior.

*H4:* Intrinsic motivation and exercise intention jointly serve as chained mediators in the relationship between fitness social media use and exercise behavior.

## 2 Materials and methods

### 2.1 Research design and participants

This study was conducted in April 2025 using a snowball sampling method. Members of the research team distributed an online electronic questionnaire targeting individuals who use social media and engage in regular physical exercise. The survey was open for 1 month. Prior to participation, respondents were given a brief overview of the general purpose of the study, although specific research questions were not disclosed. All participants provided informed consent before completing the questionnaire. A total of 467 questionnaires were collected. After excluding 27 incomplete responses and 15 questionnaires identified through manual screening as lacking response validity, 425 valid responses were retained for subsequent data analysis.

### 2.2 Instruments and measures

#### 2.2.1 Intrinsic motivation

Intrinsic motivation was measured using three subdimensions, Interest/Enjoyment, Challenge, and Competence, from the *Exercise Motivations Inventory-2* (EMI-2). Each subdimension included three items, resulting in a total of nine items. Responses were rated on a 5-point Likert scale (1 = strongly disagree, 5 = strongly agree). The EMI-2, developed by Markland and Ingledew (1997), has been widely applied in the field of exercise psychology (Markland and Ingledew, 1997). Grounded in Self-Determination Theory (SDT), the scale conceptualizes intrinsic motivation as a behavioral driver stemming from interest in the activity itself, the pursuit of challenge, and the desire for personal competence development.

Sample items included: *“I exercise because I find it enjoyable,” “I exercise to challenge my limits,”* and *“I exercise to improve my physical competence.”* The average of the total score across the nine items was calculated to represent the level of intrinsic motivation. The EMI-2 has demonstrated robust factorial and construct validity in previous studies (Ednie and Stibor, 2017), supporting its appropriateness for measuring intrinsic motivation in exercise settings. In the present study, the scale demonstrated good internal consistency (Cronbach’s *α* = 0.81; McDonald’s *ω* = 0.84).

#### 2.2.2 Exercise intention

Exercise intention was assessed based on the Theory of Planned Behavior (Ajzen, 1991), with reference to prior studies by Hagger et al. (2002). A three-item scale was used to measure participants’ intention to engage in physical activity, rated on a 5-point Likert scale (1 = strongly disagree, 5 = strongly agree).

Sample items included: *“I intend to exercise regularly over the next week,” “I plan to exercise at least three times a week,”* and *“I am determined to stick to my exercise plan in the coming weeks.”*

The average of the three items was computed to represent each participant’s level of exercise intention, with higher scores indicating stronger behavioral intention to engage in future physical activity. The scale demonstrated good internal consistency in the current study (Cronbach’s *α* = 0.88).

#### 2.2.3 Fitness social media use

Drawing upon Uses and Gratifications Theory (UGT) and Social Cognitive Theory (SCT), a scale was developed to measure fitness social media use, based on prior research by Tiggemann and Zaccardo (2015), Liu et al. (2022), and others. This scale assesses the frequency and tendency with which individuals engage with fitness-related content on social media platforms, including information acquisition, social interaction, behavioral imitation, and content sharing.

The scale comprises four dimensions with a total of 12 items: (1) Content Consumption Frequency (e.g., “I frequently browse fitness-related content on social media”), (2) Interaction Frequency (e.g., “I often like or comment on posts related to fitness”), (3) Imitation and Adoption Behavior (e.g., “I have tried to imitate fitness moves I saw on social media”), (4) Content Creation and Self-Presentation (e.g., “I share my fitness achievements on social media platforms”). All items were rated on a 5-point Likert scale (1 = strongly disagree, 5 = strongly agree). The scale was newly developed for this study based on theoretical foundations and prior empirical frameworks, the four-dimensional structure was grounded in well-established constructs from UGT and SCT. Content validity was ensured through expert review and item refinement. In the present sample, the scale demonstrated high internal consistency (Cronbach’s *α* = 0.82; McDonald’s *ω* = 0.85).

#### 2.2.4 Exercise behavior

Participants’ physical activity levels were assessed using the Physical Activity Rating Scale (PARS-3) (Yang et al., 2021). This instrument includes three items that focus on the intensity, duration, and frequency of physical activity. Each item is rated using a 5-point Likert scale. The frequency item provides the following options: “Less than once a month (1 point), 2–3 times per month (2 points), 1–2 times per week (3 points), 3–5 times per week (4 points), and daily (5 points).” The overall physical activity score was calculated using the formula: (Intensity Score, Duration Score − 1, Frequency Score), which reflects general activity level over the past month. The PARS-3 has demonstrated good psychometric properties in previous Chinese populations, including acceptable construct validity and cross-sample applicability. In the present study, the scale showed satisfactory internal consistency (Cronbach’s *α* = 0.78; McDonald’s *ω* = 0.80).

### 2.3 Statistical analysis

All statistical analyses were conducted using SPSS version 26.0. First, Harman’s single-factor test was performed to assess the potential influence of common method bias. Descriptive statistics were then calculated for all study variables. The internal consistency of each scale was evaluated using both Cronbach’s alpha (*α*), with values above 0.70 considered indicative of acceptable reliability. Given that some variables showed minor deviations from normality, Spearman’s correlation analysis was employed to examine the bivariate associations among the main constructs. To test the proposed chained mediation model, the PROCESS macro for SPSS (version 3.3) developed by Hayes (2018) was used. Model 6 was applied to assess the serial mediating roles of intrinsic motivation and exercise intention in the relationship between fitness social media use and exercise behavior. Bootstrapping procedures with 5,000 resamples were conducted to estimate 95% bias-corrected confidence intervals for the indirect effects. An indirect effect was considered statistically significant if the CI did not include zero. Effect sizes were reported using standardized regression coefficients (*β*) and coefficients of determination (*R*^2^) to evaluate the magnitude of both direct and indirect paths.

## 3 Results

### 3.1 Demographic characteristics of participants

Table 1 summarizes the demographic characteristics of the participants. The sample consisted of 226 males and 199 females. Individuals aged 26–40 accounted for 58.8% of the total sample. Over half of the respondents were married, and 50.4% held a bachelor’s degree or higher.

**Table 1:** Participants’ characteristics.

Variable	Category	Frequency	Percentage
Gender	Male	226	53.2%
	Female	199	46.8%
Age	<18	11	2.6%
	18–25	30	7.1%
	26–30	127	29.9%
	31–40	123	28.9%
	41–50	106	24.9%
	51–60	14	3.3%
	>60	14	3.3%
Education	Junior high school and below	36	8.5%
	High school	70	16.5%
	Junior college	105	24.7%
	Undergraduate	138	32.5%
	Graduate student	76	17.9%
Marital status	Single	157	36.9%
	Married	245	57.6%
	Divorced	23	5.5%

### 3.2 Common method bias test

Harman’s single-factor test was conducted to examine the presence of common method bias in the survey data. This technique involves performing an exploratory factor analysis on all questionnaire items to assess whether a substantial amount of variance can be attributed to a single factor, which would indicate potential bias due to common method variance. The unrotated principal component analysis revealed four distinct factors, with the first (largest) factor accounting for 38.58% of the total variance—below the commonly accepted threshold of 40%. These results suggest that common method bias is not a serious concern in the present study.

### 3.3 Correlation analysis among key variables

Correlation analyses revealed that all key variables were significantly associated with one another. Specifically, exercise behavior was moderately and positively correlated with fitness social media use (*r* = 0.436, *p* < 0.01), and was also significantly positively correlated with intrinsic motivation (*r* = 0.390, *p* < 0.01) and exercise intention (*r* = 0.404, *p* < 0.01). In addition, fitness social media use was significantly positively correlated with intrinsic motivation (*r* = 0.406, *p* < 0.01) and exercise intention (*r* = 0.409, *p* < 0.01). A significant positive correlation was also observed between intrinsic motivation and exercise intention (*r* = 0.407, *p* < 0.01) (Table 2). These results indicate strong interrelationships among the core variables, providing a solid empirical foundation for subsequent model testing and hypothesis validation.

**Table 2:** Correlation analysis among key variables.

	Mean	SD	EB	FSMU	IM	EI
EB	29.61	27.69	1			
FSMU	36.91	9.86	0.436**	1		
IM	30.89	7.91	0.390**	0.406**	1	
EI	10.09	2.86	0.404**	0.409**	0.407**	1

EB, Exercise Behavior; FSMU, Fitness Social Media Use; IM, Intrinsic Motivation; EI, Exercise Intention.

### 3.4 Mediation analysis

**Figure 2:**
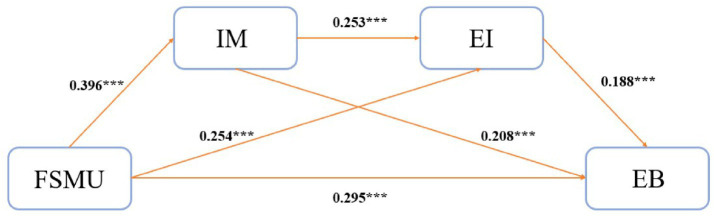
Final model showing standardized regression coefficients between fitness social media use and exercise behavior. ***p < 0.001. FSMU, Fitness Social Media Use; IM, Intrinsic Motivation; EI, Exercise Intention; EB, Exercise Behavior.

According to the results of the mediation analysis (Table 3), fitness social media use significantly and positively predicted intrinsic motivation (*β* = 0.396, *t* = 8.862, *p* < 0.001), exercise intention (*β* = 0.254, *t* = 5.272, *p* < 0.001), and exercise behavior (*β* = 0.295, *t* = 6.323, *p* < 0.001). Meanwhile, intrinsic motivation was found to significantly predict both exercise intention (*β* = 0.253, *t* = 5.254, *p* < 0.001) and exercise behavior (*β* = 0.208, *t* = 4.462, *p* < 0.001). Additionally, exercise intention significantly predicted exercise behavior (*β* = 0.188, *t* = 4.106, *p* < 0.001). The overall model demonstrated good explanatory power, with R^2^ values of 0.158, 0.180, and 0.282 for the three respective models. All *F*-values were statistically significant (*p* < 0.001), indicating that the model offers a satisfactory level of explanatory adequacy (Figure 2).

**Table 3:** The relationship between fitness social media use and exercise-related psychological and behavioral outcomes.

Item	IM		EI		EB	
	*β*	*t*	*β*	*t*	*β*	*t*
Gender	−0.007	−0.158	−0.004	−0.093	−0.015	−0.351
Age	0.014	0.306	0.020	0.459	−0.053	−1.263
FSMU	0.396	8.862***	0.254	5.272***	0.295	6.323***
IM			0.253	5.254***	0.208	4.462***
EI					0.188	4.106***
*R*	0.397		0.425		0.531	
*R* ^2^	0.158		0.180		0.282	
*F*	26.295***		23.100***		32.880***	

EB, Exercise Behavior; FSMU, Fitness Social Media Use; IM, Intrinsic Motivation; EI, Exercise Intention.

### 3.5 Mediation path analysis

To further examine the mediating effects, a bootstrap analysis was conducted with 5,000 resamples and a 95% confidence interval (Alfons et al., 2022). The analysis assessed the total effect, direct effect, and indirect effects of fitness social media use on exercise behavior. The results showed that the total effect of social media use on exercise behavior was 1.2457, with a 95% confidence interval of [1.0046, 1.4868], which does not include zero, indicating a statistically significant total effect. The direct effect was 0.8275 (95% CI [0.5703, 1.0847]), and the indirect effect was 0.4182 (95% CI [0.293, 0.5497]), accounting for approximately 33.55% of the total effect—demonstrating a meaningful mediation effect. Specifically, the first mediation path (via intrinsic motivation) had an effect size of 0.2314 (95% CI [0.1306, 0.341]), accounting for 18.57% of the total effect. The second mediation path (via exercise intention) showed an effect size of 0.1339 (95% CI [0.059, 0.2184]), representing 10.75% of the total effect. The third mediation path (via both intrinsic motivation and exercise intention in sequence) yielded an effect of 0.0529 (95% CI [0.0225, 0.0919]), contributing 4.25% to the total effect. These findings suggest that the first two mediation paths play a more prominent role in the mechanism by which social media use influences exercise behavior. Furthermore, the comparison between different indirect paths was also statistically significant (C2 = 0.1786, 95% CI [0.0642, 0.2957]), providing additional support for the robustness of the proposed mediation model (Table 4).

**Table 4:** Bootstrapped mediation effects of intrinsic motivation and exercise intention between fitness social media use and exercise behavior.

Impact pathways	Effect	BootSE	BootLLCI	BootULCI	Effect size ratio
Total effect	1.2457	0.1227	1.0046	1.4868	
Direct effect	0.8275	0.1309	0.5703	1.0847	
Total indirect effect	0.4182	0.0655	0.293	0.5497	33.55%
Ind1: FSMU → IM → EB	0.2314	0.054	0.1306	0.341	18.57%
Ind2: FSMU → EI → EB	0.1339	0.0403	0.059	0.2184	10.75%
Ind3: FSMU → IM → EI → EB	0.0529	0.0177	0.0225	0.0919	4.25%
C1 (FSMU → IM → EB − FSMU → EI → EB)	0.0976	0.073	−0.047	0.2415	
C2 (FSMU → IM → EB − FSMU → IM → EI → EB)	0.1786	0.0593	0.0642	0.2957	
C3 (FSMU → EI → EB − FSMU → IM → EI → EB)	0.081	0.0376	0.0135	0.1613	

Bootstrapped standard errors (BootSE) and 95% confidence intervals (BootLLCI, BootULCI) are based on 5,000 resamples. Ind1, via Intrinsic Motivation; Ind2, via Exercise Intention; Ind3, via Intrinsic Motivation → Exercise Intention. Effect size ratio indicates the proportion of the total effect explained by each indirect path.

## 4 Discussion

The present study aimed to explore the pathways through which fitness social media use influences individuals’ exercise behavior, with a particular focus on the mediating roles of intrinsic motivation and exercise intention. The findings revealed that fitness social media use positively predicts exercise behavior and that both intrinsic motivation and exercise intention serve as significant mediators in this relationship. All three mediation paths were statistically significant, and the chained mediation model was supported. Notably, the indirect effects accounted for 33.55% of the total effect, indicating that a substantial portion of social media’s influence on exercise behavior operates through its capacity to stimulate individuals’ motivation and intention to engage in physical activity.

### 4.1 The direct effect of fitness social media use on exercise behavior

This study found that fitness social media use significantly and positively predicts individuals’ exercise behavior. Specifically, individuals who frequently consume, engage with, or contribute to fitness-related content on social media tend to report higher levels of physical activity (*β* = 0.295, *p* < 0.001). This finding aligns with previous studies and reinforces the role of social media not only as a health information source but also as an active driver of behavioral change (Ghahramani et al., 2022; Korda and Itani, 2013; Yoo et al., 2018). By shaping group identity and establishing health-related social norms, fitness-oriented platforms provide a psychosocial environment conducive to sustained engagement in exercise.

Unlike traditional health communication channels, social media offers richer forms of interaction, including passive content consumption (watching workout videos) (Johnston and Davis, 2019; Mulchrone, 2021), active engagement (liking, commenting, or participating in challenges) (Khan, 2017; Shahbaznezhad et al., 2021), and self-presentation (sharing personal fitness progress) (Hollenbaugh, 2021; Kim, 2024). These digital interactions contribute to a media space characterized by situational immersion and social feedback, which together help reduce the psychological cost of initiating physical activity. This interpretation is supported by earlier evidence showing that media environments rich in visual stimulation and peer interaction increase the likelihood of health-related behavior adoption (Balatsoukas et al., 2015; Odenigbo et al., 2022). The current findings extend this view by demonstrating that fitness social media not only delivers content but also generates motivational resonance.

Furthermore, the findings are consistent with Bandura’s concept of an “enabling environment,” which posits that individuals exposed to consistent modeling, social cues, and positive reinforcement are more likely to reduce behavioral uncertainty and increase action motivation (Bandura, 2003; Bandura et al., 2003). As individuals become increasingly immersed in and emulate fitness-related content, they tend to internalize health-related values, shifting from cognitive attention to actual behavioral engagement. This process aligns with established theories in media influence and behavior change (Bellg, 2003; Conner and Norman, 2015; Houlihan, 2018; Renner and Schwarzer, 2003). Therefore, the direct effect of fitness social media use on exercise behavior reflects not only information acquisition but also the activation of motivational and normative processes embedded in digital social environments. These findings provide a foundation for developing social media–based health interventions that emphasize peer modeling, user engagement, and value reinforcement to promote physical activity.

### 4.2 The mediating role of intrinsic motivation between fitness social media use and exercise behavior

The findings indicate that intrinsic motivation plays a significant mediating role in the relationship between fitness social media use and exercise behavior (Liu et al., 2023; Pham Thi and Duong, 2025). This suggests that the influence of social media on physical activity is not solely direct, but also largely dependent on its capacity to stimulate individuals’ internal psychological drives (Gerber et al., 2025; Stults-Kolehmainen, 2023). Frequent engagement with fitness-related information, interactions, and content imitation on social media enhances individuals’ interest, enjoyment, and sense of challenge associated with exercise, thereby fostering stronger intrinsic motivation, which in turn facilitates behavioral engagement (González-Serrano et al., 2024; Rowles, 2021).

This mechanism aligns with the core assumptions of Self-Determination Theory (SDT), which emphasizes that intrinsic motivation, rooted in autonomy and genuine interest, is essential for sustaining long-term behavioral change (Ryan and Deci, 2000b). Within the social media fitness ecosystem, individuals are not passive consumers of health information but active agents in shaping their motivational landscape. Through engaging with training content, following fitness influencers, and participating in online challenges, users gradually internalize a sense of enjoyment and personal identification with physical activity. This internalized drive is more durable than extrinsic motivations based on rewards or pressure (Ryan, 1982; Ryan and Deci, 2017; Ryan and Deci, 2000a).

Prior studies have shown that intrinsic motivation is not only a critical antecedent to exercise maintenance but also positively associated with exercise frequency and adherence (Rodrigues et al., 2020b; Teixeira et al., 2012). The current study extends these findings by demonstrating that social media platforms can serve as environments that cultivate intrinsic motivation, thereby reinforcing health behavior through emotional and cognitive engagement. Unlike externally driven interventions, media strategies that prioritize intrinsic motivational factors, such as enjoyment, autonomy, and self-challenge, may offer greater sustainability and user commitment. Practically, this highlights the potential for social media–based health campaigns to enhance intrinsic motivation by designing content that is enjoyable, achievement-oriented, and psychologically engaging (Dera, 2024; Zhang et al., 2019). Rather than focusing solely on behavioral outcomes, future interventions should prioritize motivational processes that support the internalization of healthy behaviors. These findings contribute to a growing body of evidence affirming the centrality of psychological engagement in effective digital health promotion.

### 4.3 The mediating role of exercise intention between fitness social media use and exercise behavior

This study further revealed that exercise intention also serves as a significant mediator in the relationship between fitness social media use and exercise behavior, providing additional support for the role of cognitive regulation mechanisms in the development of health behaviors. In the context of social media, individuals who are frequently exposed to fitness content gradually form more positive attitudes toward exercise, experience greater perceived behavioral control, and perceive stronger social support and normative expectations from others. These elements collectively foster a forward-looking psychological orientation marked by internal commitments such as “I will start working out” or “I should stay consistent with my routine” (Carpenter and Amaravadi, 2019; Conner et al., 2010; Korda and Itani, 2013; Zhang et al., 2015). This forward-looking and goal-oriented cognitive state enhances the likelihood of behavioral execution.

As a critical bridge between cognition and action, exercise intention has been widely validated as a strong predictor of health behavior across numerous studies (Hagger et al., 2002). The current findings confirm the applicability of this mechanism within the social media environment, showing that social media exerts an indirect influence on exercise behavior by shaping individuals’ behavioral expectations and plans (Lutkenhaus et al., 2023; Vaterlaus et al., 2015). The formation of such intentions is not only driven by motivational levels but also shaped by social influences embedded in the platform, such as social comparison, role modeling, and peer pressure, which collectively transform behavioral possibilities into behavioral tendencies (Contractor and DeChurch, 2014; Garcia et al., 2013).

It is noteworthy that, compared to intrinsic motivation, exercise intention is cognitively closer to the behavior itself and more strongly execution-oriented (Godin et al., 1986; Yordy and Lent, 1993). This distinction provides a critical insight for intervention design: while stimulating motivational desire is essential, ensuring that motivation is translated into actionable plans is equally vital. Therefore, effective health promotion strategies on social media should simultaneously address motivational appeal and facilitate goal setting, implementation intentions, and behavioral reinforcement mechanisms (Albalawi and Sixsmith, 2015; Ghahramani et al., 2022; Korda and Itani, 2013). Furthermore, the present study helps explain the often-observed phenomenon that “high motivation does not necessarily lead to high action,” highlighting that the relationship between motivation and behavior is not linear, and that intention serves as a crucial mediating factor in this process.

### 4.4 The chain mediation effect of intrinsic motivation and exercise intention

This study confirmed the chain mediation effect of intrinsic motivation and exercise intention in the relationship between fitness social media use and exercise behavior. The findings suggest that the influence of social media on individuals’ exercise behavior unfolds as a sequential psychological process, from emotional activation to cognitive planning and finally to behavioral execution. Specifically, exposure to fitness-related content on social media stimulates individuals’ interest and desire for challenge, thereby enhancing their intrinsic motivation. On this basis, individuals are more likely to form clear behavioral intentions, which in turn promote actual engagement in exercise (Durau et al., 2022; Mulchrone, 2021; Rowles, 2021).

These findings offer robust support for the theoretical integration of Self-Determination Theory (SDT) and the Theory of Planned Behavior (TPB) in explaining the psychological mechanisms underlying health behavior change. While SDT highlights intrinsic motivation as the internal driver of voluntary behavior (Deci and Ryan, 2013; Deci and Ryan, 1985; Ryan and Deci, 2000b), TPB emphasizes behavioral intention as the proximal antecedent of action (Ajzen, 1985; Ajzen, 1991). By empirically validating a sequential pathway from motivation to intention to behavior, the current model bridges these two frameworks, providing a more comprehensive explanation of how digital media influence real-world behavior. This integrative approach addresses the limitations of single-theory models and illuminates the layered psychological processes activated by social media engagement.

From a practical standpoint, the chain mediation pathway underscores the need for health interventions that target both emotional activation and cognitive planning. Strategies that focus solely on emotional resonance—such as motivational messages or inspirational content, may generate short-term interest but fall short in fostering sustained action (Brand and Ekkekakis, 2021; Van Cappellen et al., 2018). Instead, the key lies in supporting individuals to transform motivational states into clear behavioral intentions, such as through goal setting, progress tracking, or commitment prompts. By emphasizing both the “why” and the “how” of behavior change, social media interventions can more effectively convert user engagement into lasting exercise habits. Ultimately, this study advances both theoretical and applied understanding of how digital environments shape health behavior through interconnected motivational and intentional processes.

## 5 Limitations

This study presents several limitations that should be acknowledged. The cross-sectional nature of the data limits the ability to infer causality, as the proposed mediation pathways reflect associations rather than directional effects. Future research employing longitudinal or experimental designs could help clarify the temporal sequencing and strengthen causal claims (Maxwell and Cole, 2007; Selig and Preacher, 2009). In addition, the reliance on self-report measures may introduce potential bias, particularly in reporting exercise behavior, where participants may overestimate frequency or intensity (Prince et al., 2008). Integrating objective indicators such as wearable device data or digital activity logs could improve measurement validity. The sample also reflects limited diversity in age and cultural background, which may restrict the generalizability of the findings. Expanding the participant pool across cultural contexts and social media platforms would offer deeper insights into the universality or specificity of the observed relationships. While this study focused on intrinsic motivation and exercise intention as mediators, other psychological constructs, such as self-efficacy, social identity, or body image, may also play meaningful roles and warrant further exploration.

## 6 Conclusion

This study proposed and validated a chained mediation model to examine how fitness social media use influences exercise behavior through intrinsic motivation and exercise intention. The results indicate that fitness social media use not only directly promotes exercise behavior but also exerts an indirect effect by enhancing intrinsic motivation and shaping behavioral intention. All three mediation paths were significant, and the chain mediation effect involving intrinsic motivation and exercise intention was supported, providing new insight into the psychological transformation process from media exposure to actual exercise engagement. These insights contribute to a deeper theoretical understanding of how digital platforms influence health behavior and offer a practical foundation for promoting sustained exercise. By targeting both intrinsic motivation and behavioral intention, social media interventions can be more effective in translating user engagement into lasting physical activity. These findings can help educators and fitness coaches design more psychologically informed and goal-oriented interventions in digital health promotion.

## Data Availability

The raw data supporting the conclusions of this article will be made available by the authors without undue reservation.
